# Auditory Discrimination Learning: Role of Working Memory

**DOI:** 10.1371/journal.pone.0147320

**Published:** 2016-01-22

**Authors:** Yu-Xuan Zhang, David R. Moore, Jeanne Guiraud, Katharine Molloy, Ting-Ting Yan, Sygal Amitay

**Affiliations:** 1 Medical Research Council—Institute of Hearing Research, University Park, Nottingham, United Kingdom; 2 State Key Laboratory of Cognitive Neuroscience and Learning & IDG/McGovern Institute for Brain Research, Beijing Normal University, Beijing, China; University of Akron, UNITED STATES

## Abstract

Perceptual training is generally assumed to improve perception by modifying the encoding or decoding of sensory information. However, this assumption is incompatible with recent demonstrations that transfer of learning can be enhanced by across-trial variation of training stimuli or task. Here we present three lines of evidence from healthy adults in support of the idea that the enhanced transfer of auditory discrimination learning is mediated by working memory (WM). First, the ability to discriminate small differences in tone frequency or duration was correlated with WM measured with a tone n-back task. Second, training frequency discrimination around a variable frequency transferred to and from WM learning, but training around a fixed frequency did not. The transfer of learning in both directions was correlated with a reduction of the influence of stimulus variation in the discrimination task, linking WM and its improvement to across-trial stimulus interaction in auditory discrimination. Third, while WM training transferred broadly to other WM and auditory discrimination tasks, variable-frequency training on duration discrimination did not improve WM, indicating that stimulus variation challenges and trains WM only if the task demands stimulus updating in the varied dimension. The results provide empirical evidence as well as a theoretic framework for interactions between cognitive and sensory plasticity during perceptual experience.

## Introduction

Perceptual learning, the improvement of perception with experience, is typically specific to the trained task and stimuli and rarely transfers to untrained ones [1, 2]. The specificity of learning is not only a practical concern, as it heavily limits the benefits of perceptual training, but also a theoretical one, as it has been used to infer the stage of neural processing where learning occurs [3] or the information-weighting strategies learned at the decision-making level [4, 5]. A series of studies has suggested that learning specificity can be influenced by the variability of training materials [6, 7]. Conventionally, perceptual learning has been experimentally induced via repetitive training on a single task performed using a single standard stimulus. In such cases, all training trials are identical except for small variations in the task-relevant stimulus dimension, along which participants are asked to make discrimination or identification judgments (e.g. discriminating tone frequencies from a standard of 1 kHz). Varying the task or the standard value across trials has been reported to disrupt learning [8, 9]. However, beneficial effects of across-trial variation of training materials in terms of learning transferability have also been found [7, 10, 11]. For example, tone frequency discrimination (FD) learning takes longer, but is more transferable when the standard frequency is randomized among five values (roved) from trial to trial than when it is fixed [10]. Temporal-interval discrimination training, which does not benefit FD performance when conducted alone, does so when mixed with FD training [11]. These observations echo earlier reports of speech learning that high-variability training (e.g., speech utterances produced by multiple compared to a single talker) facilitates transfer to untrained materials [12–14].

The beneficial effects of across-trial variation during training pose challenges to existing models of perceptual learning [6]. For example, the Reverse Hierarchy Theory [3] postulates that learning occurs at the highest level along the hierarchy of sensory encoding pathways that provides sufficient signal-to-noise ratio for performing the training task. The level of sensory encoding at which learning takes place is reached by an attention driven, top-down search. Varying the task or the standard stimulus during training may disrupt learning as different training conditions require different search routes so that a consistent search strategy may not be established. Similarly, models assuming learning occurs in the decoding or ‘readout’ of sensory information also lack mechanisms to account for increased transferability caused by training multiple stimuli or tasks. For example, Lu and colleagues [4] proposed that perceptual learning resulted from Hebbian reweighting of the connections between a decision-making unit and early sensory channels, such as visual cortical neurons tuned to different orientations. Training increases weights of relevant channels and decreases weights of irrelevant channels, resulting in improved perceptual performance. According to this model, when multiple training stimuli or tasks are used, the relevance of sensory channels for perceptual performance would fluctuate, decreasing efficiency of the reweighting process. Current models thus respond adversely to variability in training, regardless of whether they attribute learning to changes in encoding or decoding of sensory information.

One candidate for mediating the beneficial effect of training variability is working memory (WM). WM is a capacity-limited system for temporarily maintaining information in a readily accessible state to support mental processing [15]. As auditory stimuli are nearly always sequentially presented, WM is indispensable for auditory processing. WM representations are known to be susceptible to interference from previous memories [15–17], thus allowing interaction of stimuli across trials. Improvement of WM during perceptual training would make it possible for learning to benefit from mixed training of different conditions.

Here we present and test a specific proposal of how WM mediates the effect of across-trial stimulus variation in auditory discrimination. When the standard stimulus varies across trials, WM has to ‘update’ memory representations at each trial. New stimuli are stored and used for judgments and outdated memories are relinquished. Imperfect updating would result in interference between current and outdated memories, compromising perceptual performance. In contrast, when the same standard stimulus is used across trials, imperfect updating has no adverse effect. Instead, existing memory provides access to multiple samples of the same standard. These may be utilized to form a more accurate representation than can be attained from a single stimulus presentation. This advantage of stimulus repetition afforded by WM is a candidate underpinning mechanism for the concept of a “perceptual anchor” [18, 19], which refers to an internal reference that is more stable and less noisy than novel sensations.

The proposed role of WM in auditory discrimination makes three specific predictions. First, discrimination performance should correlate with WM updating capacity because discrimination relies on comparison with WM representations of the standard stimulus. In the conventional case of having a constant standard, WM updating results in a stable ‘perceptual anchor’. Varying the standard stimulus should reduce performance by introducing interference between memory representations, but only if those representations are used for discrimination judgments (i.e. the variation is along the to-be-discriminated stimulus dimension). Performance differences between discrimination with a varying and a constant standard could constitute an index for WM interference. Second, because discrimination depends on WM representations, improved WM should enhance auditory discrimination. Specifically, improvements in the ability to resist interference should benefit auditory discrimination with a varying, but not with a constant standard stimulus, because memory interferences compromise performance in the former but not the latter case. Third, the WM processes involved in auditory discrimination are, like sensory encoding and decoding, subject to modification by perceptual training. Accordingly, WM may improve with perceptual training under conditions in which WM processes limit discrimination performance. Specifically, training discrimination with the standard varying in the task relevant dimension should engage WM updating processes and may thus improve WM. In contrast, training discrimination with a constant standard, or with stimulus variations irrelevant to the training task, is unlikely to challenge and hence to improve WM updating. Some of these predictions receive limited support from existing literature. For example, varying (‘roving’) the standard frequency during FD training has been found to elevate FD threshold relative to a constant standard frequency [10], indicating that memory interference compromises discrimination performance. Further, dyslexic children with WM impairments reportedly fail to form a perceptual anchor [19], even under conditions that promote this strategy (using constant standards), suggesting that the ‘perceptual anchor’ also depends on WM. The current study presents a systematic examination of these three predictions in the context of learning and transfer. The results of multiple training experiments support the proposal that WM mediates the effect of across-trial stimulus variation in auditory training.

## Materials and Methods

### Ethics Statement

All procedures were approved by both the Nottingham University Hospitals Research Ethics Committee and the Beijing Normal University Research Ethics Committee. Participants gave written informed consent, and were compensated for their time.

### Participants

A total of 190 healthy adults aged 18–40, naive to psychoacoustic testing, were recruited from the University of Nottingham (N = 148) and Beijing Normal University (N = 42). All participants had auditory tone thresholds ≤ 20 dB HL across 0.5–4 kHz bilaterally and no known learning disorders. Participants withdrawn from the study after completing a pre-training test (N = 2) were included only in the pre-training analyses.

### Equipment

All testing and training were carried out in a double-walled sound-attenuating booth, using custom computer programs based on the Psychtoolbox for Matlab [20, 21]. Auditory stimuli were digitally generated and were presented diotically using Sennheiser HD-25-1 headphones.

### Task and Stimuli

#### Tone frequency discrimination (FD) task

**Fig 1:**
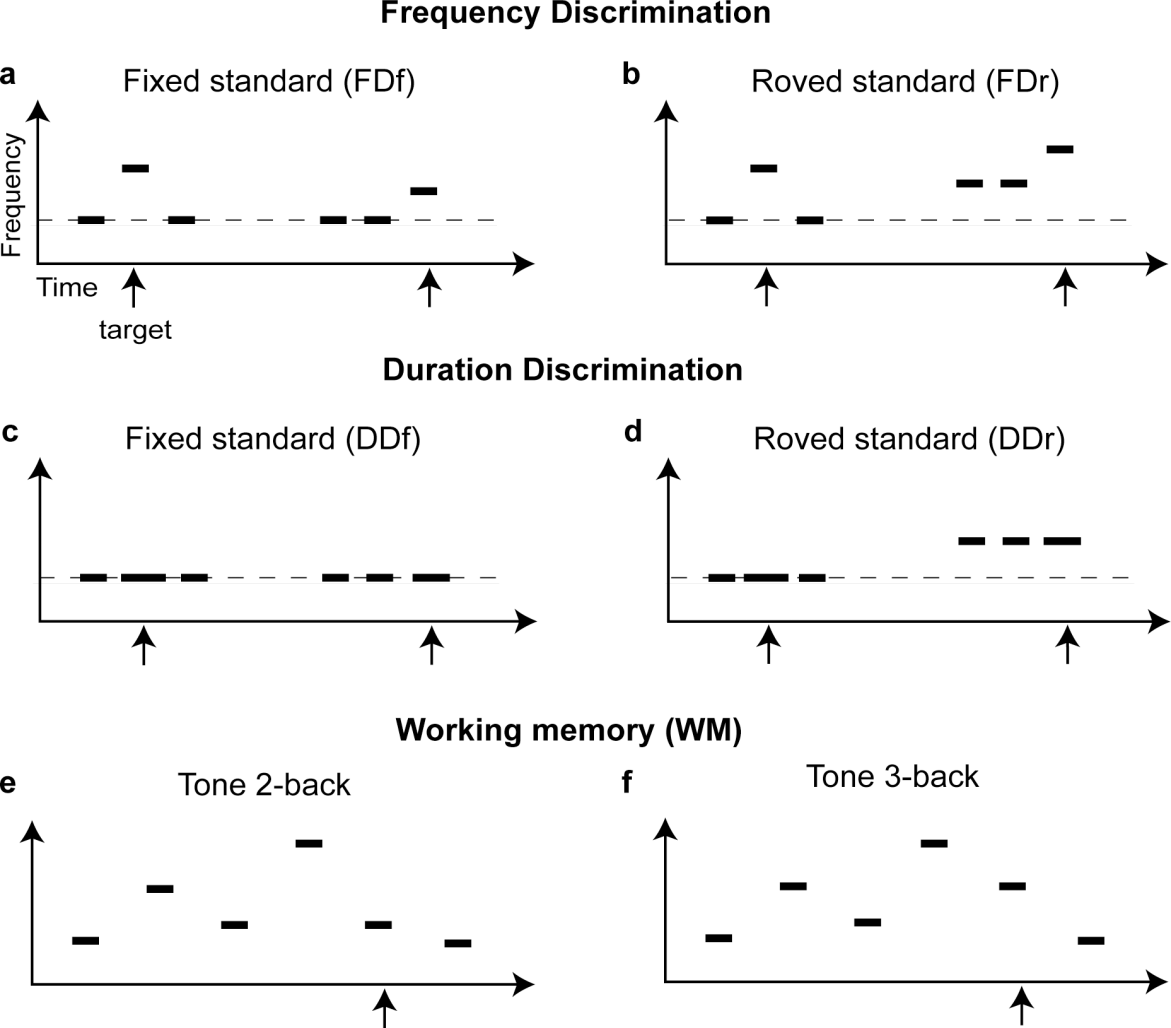
Discrimination and memory tasks. In all tasks tones (horizontal bars) were presented sequentially. The discrimination tasks required listeners to pick the different (target; marked by an arrow) stimulus from two identical (standard) stimuli. The target differed from the standards in either frequency (FD; a, b) or duration (DD; c, d) and the standard stimulus frequency was either fixed (a, FDf; c, DDf) or roved (b, FDr; d, DDr) across trials. (e, f) The Tone n-back task was used as a measure of auditory working memory. Participants compared the current tone frequency with that n positions back.

Each trial consisted of three sequentially presented tones separated by a 300-ms interstimulus interval (Fig 1A and 1B), two of which were identical (the standard) and the third had a higher frequency (the target). The serial, temporal position of the target was randomized across trials. Participants were instructed to indicate the target position by pressing one of three buttons. Feedback was provided visually after each trial. All tones were 100 ms long, including 10-ms rise-fall times, presented at 75 dB SPL. The standard tone frequency was either fixed at 1 kHz across trials (FDf; Fig 1A), or randomly chosen among 900, 950, 1000, 1050, and 1100 Hz (FDr; Fig 1B) [10].

For FD testing and training, the frequency difference between the target and the standard (∆f, expressed as percentage of the standard) was adaptively varied in blocks of 50 trials. In each block, ∆f started at 50%, and was divided (for a correct response) or multiplied (for an incorrect response) by a step size of 2 until the first reversal. Thereafter, the adaptive rule switched to 3-down 1-up, with a step size of 1.41, to estimate FD threshold at the 79% correct point on the psychometric function [22]. For example, for FD at 1 kHz, the first trial would contain two standard tones of 1 kHz and one comparison tone of 1.5 kHz (∆f = 50%). The comparison tone would become 1.25 kHz (∆f = 25%) after one correct response and become 1.125 kHz (∆f = 12.5%) after two consecutive correct responses. The ∆f would keep decreasing by a factor of 2 until the first incorrect response (the first reversal). From there on, ∆f would decrease (by a factor of 1.41 instead of 2) only after three consecutive correct responses, increase after one incorrect response, and remain unchanged otherwise.

#### Tone duration discrimination (DD) task

As for the FD task, each trial consisted of three sequentially presented tones separated by 300-ms interstimulus intervals, except that participants were asked to indicate which of the three tones had a longer duration (Fig 1C and 1D). The standard tone duration was 100 ms. The tone frequency was always fixed within a trial, but could be either fixed (DDf; Fig 1C) or roved (DDr; Fig 1D) across trials. The roved frequency values for DDr were the same as for FDr. The duration difference was adaptively adjusted in each block, initially following a 1-down-1-up rule with a starting value of 100 ms and a step of 20 ms. After the first reversal, the rule switched to 3-down-1-up, with a step size of 5 ms.

#### Working memory tasks

**n-back tasks:** We used n-back tasks to assess working memory updating [23, 24]. Participants were presented with a sequence of 40 + n items (e.g., 43 items for 3-back and 42 items for 2-back) and were instructed to press a button whenever the current item matched that presented n positions back (a target). The presentation rate was 2.5 seconds/item. No responses were required for non-targets. Twelve targets were randomly presented in the last 40 items (the first n trials are not valid testing trials and required no response). Before and during each sequence, n was displayed on the computer screen. Trial-by-Trial feedback was provided visually for auditory sequences and auditorily for visual sequences. Upon finishing a sequence, feedback on overall performance (percent correct) was also given. Performance of this task requires maintenance of at least n items in memory and constant updating of that memory. In the pre- and posttests, performance of two sequences was averaged for each n-back task.

Three types of stimuli were used with the n-back task: auditory non-verbal (tones, Fig 1E and 1F), auditory verbal (digits), and visual non-verbal (shapes). The non-verbal, tone stimuli were adapted from Gaab *et al*. [25]. All tones were 100-ms long, presented at 60 dB SPL. Each sequence used eight tone frequencies (1080 to 4022 Hz), separated by at least one equivalent rectangular bandwidth (ERB) so that the frequencies were clearly distinguishable from each other. To minimize the chance of perceptual learning, and labeling of individual tones that would enhance memory performance, eight different sets of eight frequencies were used. This allowed for a large number of tones and a small number of repetitions for each tone. The digits, recorded from a British English speaker, included the eight single-syllable digits from one to nine, with seven excluded. Each digit was of approximately the same duration and presented at 60 dB SPL. The shapes, adapted from Jaeggi *et al*. [26], were presented in yellow against a black background, positioned at the center of the screen. These shapes were designed to elude verbal description or labeling. The Tone n-back task was used for WM assessment and training in all experiments. The Digit and Shape n-back tasks were used only for testing transfer of WM learning in the WM training experiment.

**Digit Span:** We also assessed WM span using the Digit Span subtest of the Wechsler Adult Intelligence Scales (WAIS III, [27]). Participants were asked to repeat a list of spoken digits either in the order they were presented (forward recall) or in the reverse order (backward recall). Only the backward recall scores were used as a measure of verbal WM. In the WM training experiment, we tested the Digit Span both before and after training. To avoid learning from repetition, each participant was tested with a different set of digits (the other set from WAIS IV, [28]) in the pre- and post-training test, with the order of the two sets randomized across participants.

### Training

The study consisted of multiple training experiments. The specific training task and procedure are described in Results & Discussion for each experiment. A pretest—training—posttest design was used for all experiments. Training involved repetitive practice on a single task. Training sessions typically took place on consecutive days except for weekends. The pre- and post-tests included transfer tasks in addition to the training task, conducted on separate days before and after training. Control participants did not receive training, but participated in the pre- and post-tests separated by the same days of training. The testing conditions and demographic information for each group are summarized in Table 1.

**Table 1:** Demographic information for participant groups.

Study	Group	Pre- and posttest conditions	N (Female)	Mean age	Location
**FD training**	FDf training	FDf, Tone 3-back	36 (22)	21.9	UK
	FDr training	FDf, FDr, Tone 3-back	24 (15)	23.1	
	Control	FDf, FDr, Tone 3-back	24 (13)	22.8	
**WM training**	Training	All FD, DD, and WM tasks	12 (7)	27.5	UK
			6 (4)	20.8	China
	Control	All FD and WM tasks	12 (5)	21.4	China
**DDr training**	Training	DDf, DDr, Tone 3-back	9 (6)	23.8	China
	Control	DDf, DDr, Tone 3-back	15 (7)	23.2	
**WM training**^**a**^	Adaptive n	FDf, Tone 3-back	20 (8)	22.0	UK
	Fixed n	FDf, FDr, Tone 3-back	10 (6)	21.6	
	Control	FDf, Tone 3-back	20 (9)	20.3	

^a^Only pretest data are reported due to lack of WM learning

### Statistical analyses

FD thresholds (percent of standard frequency) were log-transformed for data analyses. n-back performance was measured using d’, calculated as *Z*(hit rate)–*Z*(false alarm rate), where *Z* is the inverse cumulative Gaussian distribution. Thresholds for each task before training were normally distributed (Shapiro-Wilk test of normality, alpha level at 0.05). For each condition, individuals with initial performance above or below two standard deviations of the group average were excluded from the analyses (< 3% data excluded). Learning during training was evaluated by fitting a linear regression of performance over training sessions for each training group. Transfer was indicated by group by test interaction in repeated-measures ANOVAs that compared the pre- and post-training tests between groups. For simplicity, detail statistics for group comparisons before training (Table A to D in S1 File) and main effects of group and test (Table E to H in S1 File) were presented in supporting information. Because perceptual improvements correlated with starting performance (p < 0.001), post-training thresholds were compared between groups with pre-training threshold as a covariate.

## Results and Discussion

### Pre-training relationship between WM and FD

**Fig 2:**
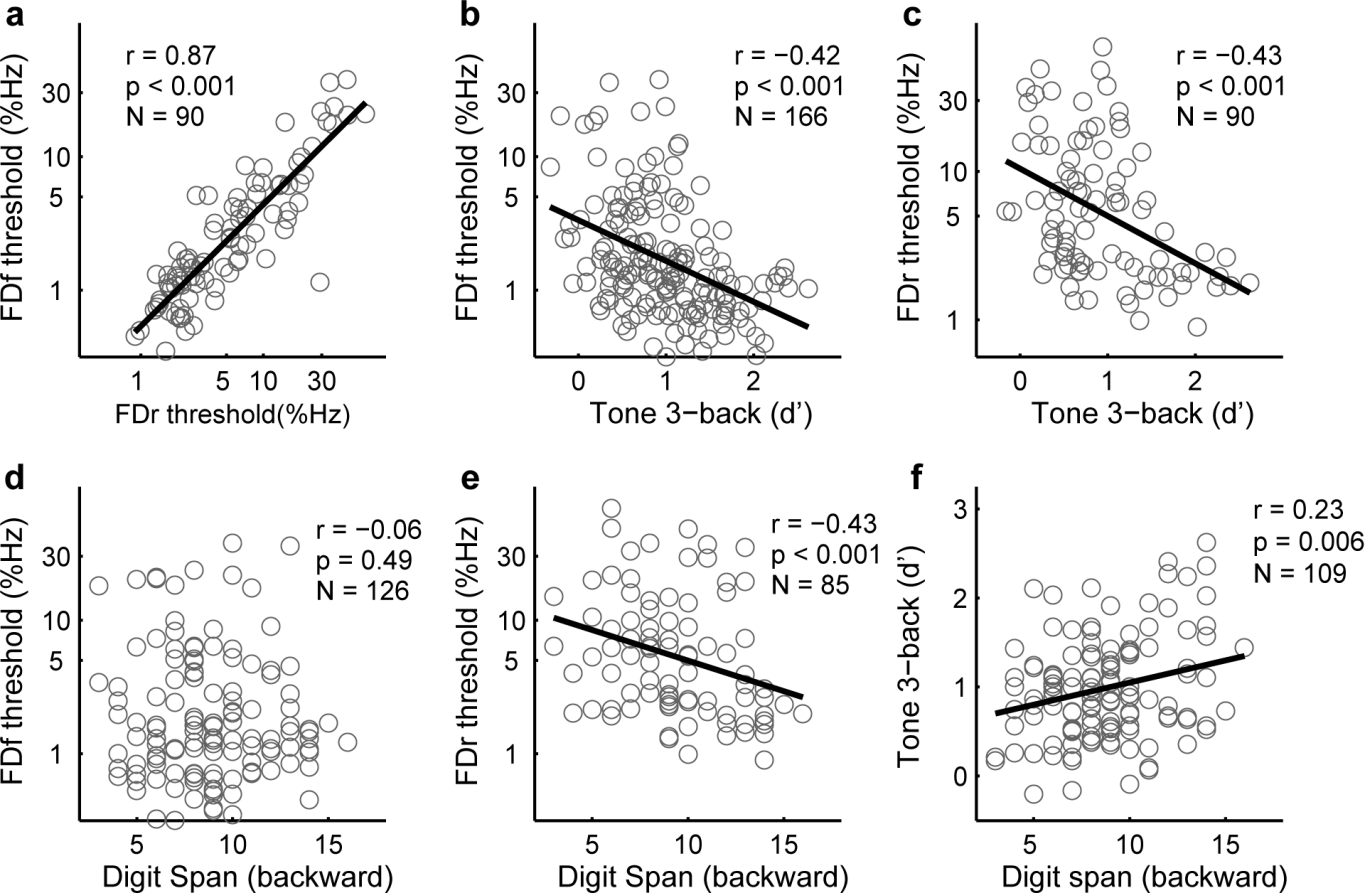
Correlations between FD and WM tasks in naïve participants. Scatterplots show the correlations between pretest measures for FDf, FDr, Tone 3-back, and backward Digit Span tasks. Sample sizes (N) are provided in each panel with correlation statistics.

Low frequency tone discrimination (FD) is a measure of auditory temporal fine structure perception [29] that is relevant to speech intelligibility in noise [30] and typically involves detecting small deviations from a standard frequency. The standard frequency can be either fixed (FDf; Fig 1A) or roved from trial to trial (FDr; Fig 1B). In common with most auditory discrimination tasks, FD requires comparison of sequentially presented sounds and thus must involve auditory memory. To test whether WM plays a performance limiting role in FD, we examined their relationship before training. Participants were tested on the two FD conditions and on two WM tasks, a Tone 3-back task assessing memory updating (Fig 1E), and a backward Digit Span task assessing maintenance and manipulation of verbal information (mean ± standard deviation for FDf: 0.23 ± 0.45 log_10_(%); FDr: 0.73 ± 0.46 log_10_(%); Tone 3-back: 0.98 ± 0.59; Digit Span: 8.8 ± 2.9). As expected, performance on the two FD tasks was significantly correlated (Fig 2A). Both FDf (Fig 2B) and FDr (Fig 2C) thresholds were significantly correlated with sensitivity (d’) on the Tone 3-back. Consistent with these correlations, higher-WM participants (those with d’ higher than mean) were better on FD than lower-WM participants (ANOVA group factor for FDf: F_1,164_ = 22.0, p < 0.001; FDr: F_1,88_ = 13.0, p = 0.001). FDf thresholds did not correlate with Digit Span scores (Fig 2D), but FDr thresholds did (Fig 2E). Interestingly, the two WM tasks were only weakly correlated (Fig 2F).

The weak correlation between the Tone n-back and backward Digit Span is consistent with previous reports [31–33] suggesting that these two WM tasks measure only partially overlapping cognitive capacities. In the current context, the WM skills measured by Tone n-back are apparently of greater relevance to FD performance than those measured by backward Digit Span.

### Training FDr improved WM updating

For most listeners, FD thresholds are much lower with a fixed than with a roved standard [10]. FDr learning of these listeners is also more protracted than FDf and transfers to FDf, but FDf learning does not transfer to FDr [10]. These observations suggest at least partially different mechanisms for FD performance and learning. A direct consequence of roving the standard is that frequencies of recent tones have to be retained in WM and updated each trial against the intrusion of memories from previous stimulus presentations. In contrast, a fixed standard places less demand on memory updating and may allow formation of a stable, refined memory representation of the standard (a 'perceptual anchor'; [18]). This hypothesized role of WM in FD predicts that training FDr improves WM updating, while training FDf may not.

**Fig 3:**
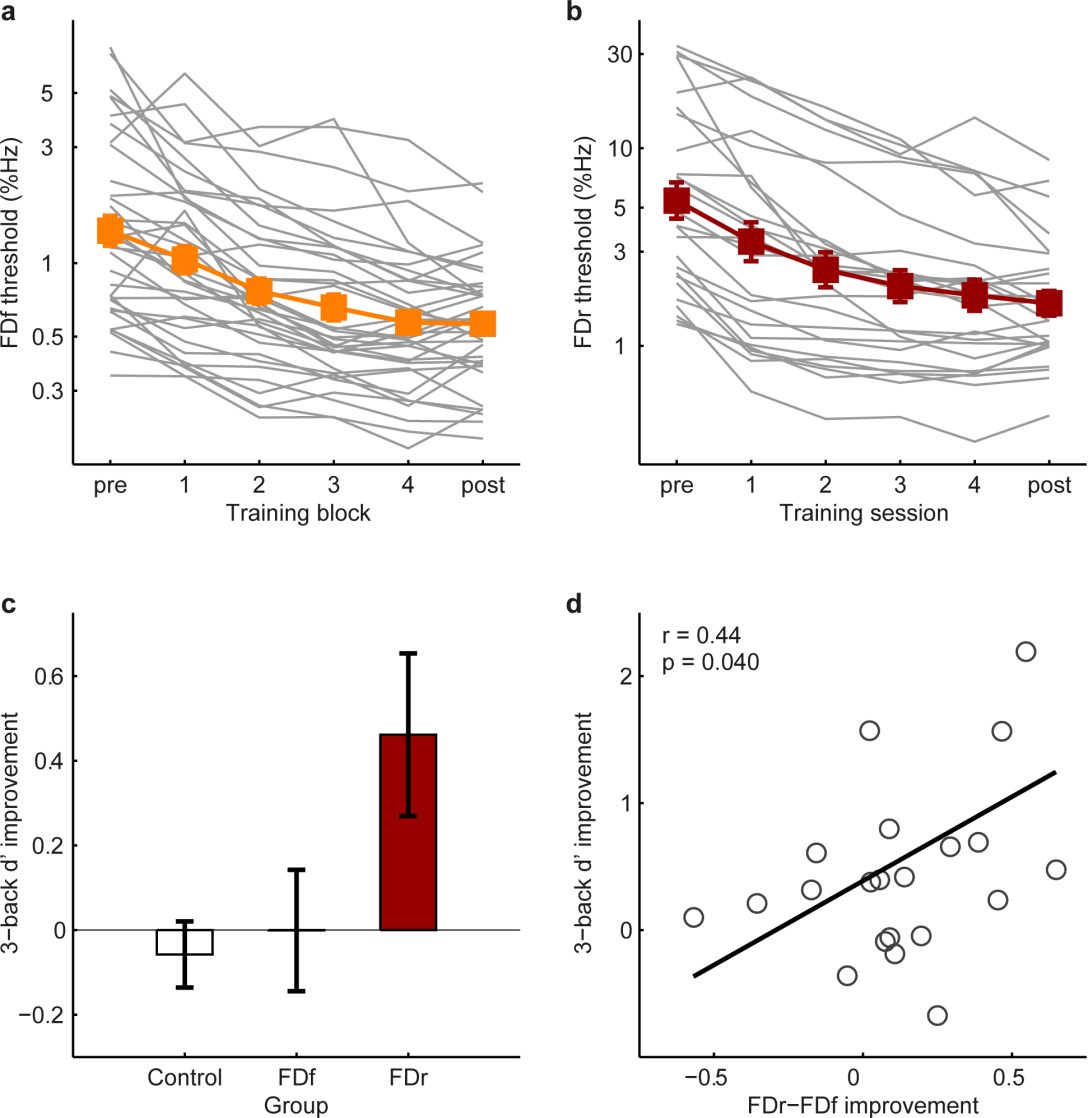
FDr but not FDf training transferred to WM. (a) FDf performance during training (N = 36) for individuals (grey lines) and group average (filled squares). (b) FDr performance during training (N = 24). (c) Improvement of FD trained and control groups on the Tone 3-back task, calculated as the improvement ratio of d’ [(posttest–pretest)/pretest]. (d) Correlation between WM improvement and FDr–FDf improvement (see text). Error bars here and in Figs 4–6 are ± 1 s.e.m.

To test this prediction, two groups of participants were trained for multiple sessions on either FDf (N = 36) for 1600 trials or FDr (N = 24) for 3600 trials. The amount of FDr training was determined based on a previous study [10] and was evenly distributed across 4 daily training sessions (~25 min/session). The FDf training participants completed training in 2, 4, or 8 daily sessions respectively (N = 12 per group). They were recruited in a previous study [34]. As pilot analyses showed no group differences in learning or transfer, we pooled together the three FDf training groups and divided their total training evenly into four blocks of 400 trials to illustrate the progress of learning (Fig 3A). Thirty six untrained participants served as controls (12 of whom were recruited for the subsequent WM training experiment, Table 1). Before training, there was no performance difference among the three groups on any of the three tasks (see Table A in S1 File).

The amount of training on each condition was sufficient for the majority of participants to reach asymptotic performance (see Fig 3A and 3B). FD threshold on both conditions improved significantly with training (FDf: F_5,175_ = 65.6, p < 0.001, FDr: F_5,125_ = 55.2, p < 0.001). Both the FDf (repeated-measures ANOVA, group by test interaction: F_1,70_ = 42.0, p < 0.001) and the FDr (F_1,58_ = 32.8, p < 0.001) training groups improved significantly more than the control group on their respective training conditions. The FDr group also improved more than controls on the Tone 3-back task, while the FDf group did not (Fig 3C; F_2,90_ = 4.25, p = 0.017; post hoc comparison, Tukey HSD: FDr vs. control: p = 0.028; FDf vs. control: p = 0.99; FDr vs. FDf: p = 0.032). To further illustrate the relationship between FD and WM learning, we examined correlations among the improvements of the FDr-training participants on the three tasks. While their improvement on FDr correlated with that on FDf (r = 0.49, p = 0.015) and Tone 3-back (r = 0.62, p = 0.002), FDf and Tone 3-back improvements did not correlate (r = -0.18, p = 0.39). This relationship could be summarized by a linear regression model of FDr learning as containing two separable components that transferred to FDf (t = 3.63, p = 0.002) and to WM updating (t = 5.17, p < 0.001). The transfer to WM can thus be viewed as difference between FDr and FDf improvement [(pretest FDr–posttest FDr)–(pretest FDf–posttest FDf)], which can be rewritten as reduction in the difference between FDr and FDf performance [pretest (FDr–FDf)–posttest (FDr–FDf)], an index of the influence of varying the standard (Fig 3D).

### Training WM updating transferred to FD

**Fig 4:**
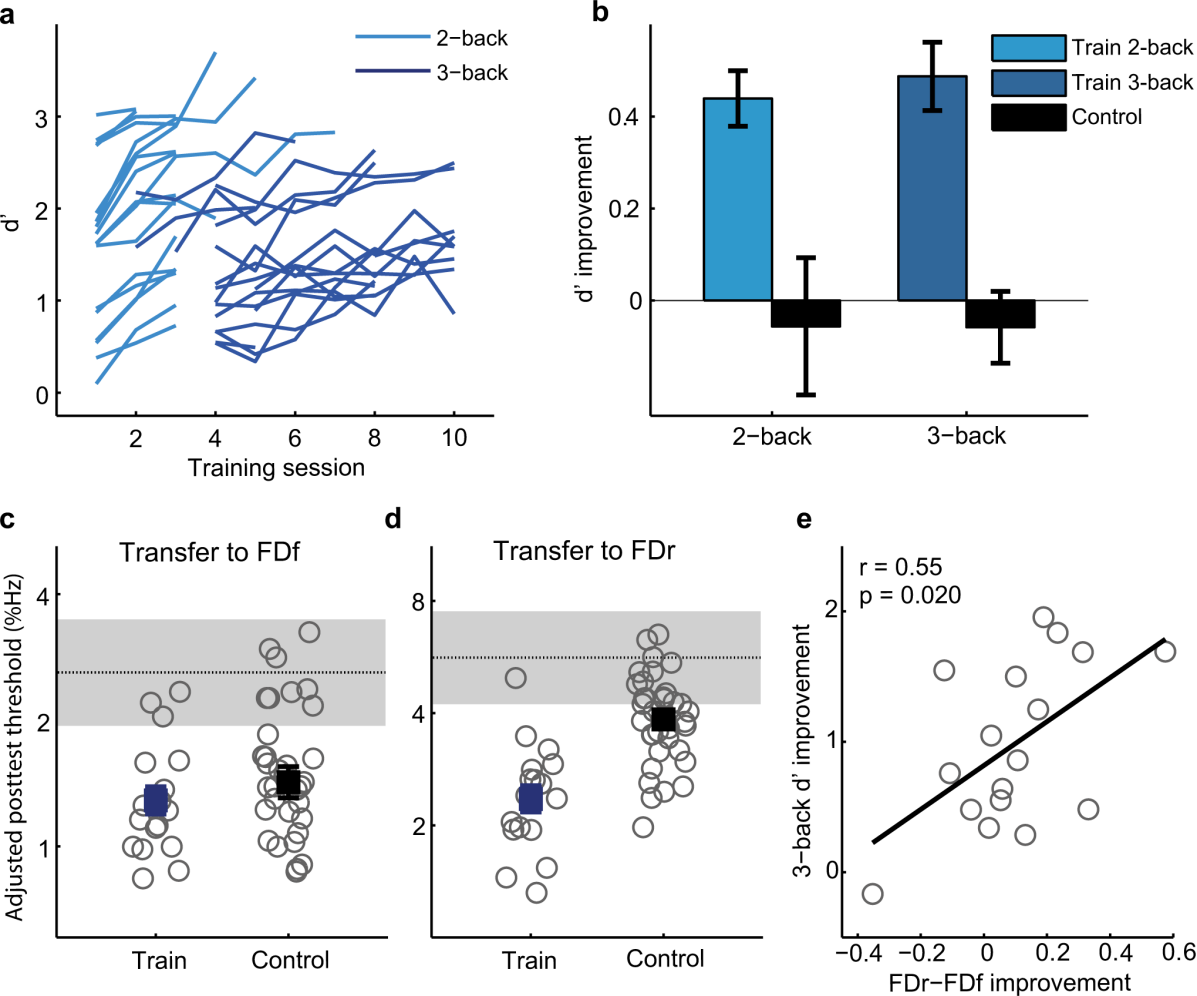
WM training and transfer to FD. (a) Multiple-session training on the Tone n-back task (N = 17), with n first fixed at 2 (light blue lines) and then 3 (dark blue lines). (b) Pre- and posttest d’ for Tone 2- and 3-back. Only twelve of the control group (N = 36) were tested on the 2-back condition. (c) Transfer to FDf. Plotted are individual (grey circles) and group mean (filled squares) posttest thresholds, adjusted for differences in pretest thresholds. The horizontal dotted line and the grey patch indicate mean pretest threshold and 95% confidence interval. (d) Transfer to FDr. (e) Correlation between WM improvement and reduction of the influence of variability (FDr–FDf) in the trained participants.

If WM updating plays a performance limiting role in FDr as we suggested, FDr performance should also benefit from improved WM updating skills. To test this, we trained a further group (N = 17) for multiple daily sessions of 10 blocks (~20 min; Fig 4A). During multiple session training, the n level was first fixed at 2 and then switched to 3, either after 2-back performance improved for three consecutive sessions or when 2-back performance exceeded 90% correct for five out of eight consecutive blocks. Training terminated when 3-back performance reached 85% correct for five out of eight consecutive blocks, or after the 10^th^ session. This flexible training regimen was intended to encourage learning, as in pilot studies two groups trained on Tone n-back with n either adaptively changed or fixed at 3 failed to improve. Before training, there was no difference between the training and the control groups in FD or WM performance (see Table B in S1 File).

Both 2- and 3-back performance improved significantly with training (Fig 4A; 2-back: F_1,11_ = 23.57, p = 0.001; 3-back: F_1,11_ = 26.12, p < 0.001) and more than controls between the tests (Fig 4B; 2-back: F_1,27_ = 10.74, p = 0.003; 3-back: F_1,51_ = 53.01, p < 0.001). Despite the preceding 2-back training, 3-back performance in the first training block did not differ from that in the pretest (paired t-test, t_11_ = 0.11, p = 0.91), suggesting that WM learning is load specific. The trained group improved more than controls for the FDr condition (Fig 4D; F_1,50_ = 19.59, p < 0.001), indicating transfer from WM training to perceptual performance. However, the WM training failed to improve FDf performance beyond the test-retest learning of controls (Fig 4C; F_1,49_ = 0.05, p = 0.82), which was significant (paired sample t-test: t_33_ = 6.65, p < 0.001). Tone 3-back improvement was correlated with reduction in the influence of stimulus variability (FDr–FDf; Fig 4E), paralleling the results of FDr training (Fig 3D).

### Transfer of WM learning to other tasks

One alternative explanation of the mutual transfer between FDr and Tone n-back is that the transfer is mediated by the superficial similarity of the two tasks. As both tasks employ pure tones of variable frequencies and engage judgments on the frequency dimension, listeners might have simply become familiar with this particular type of stimuli, or have learned to direct attention more readily to sound frequency.

**Fig 5:**
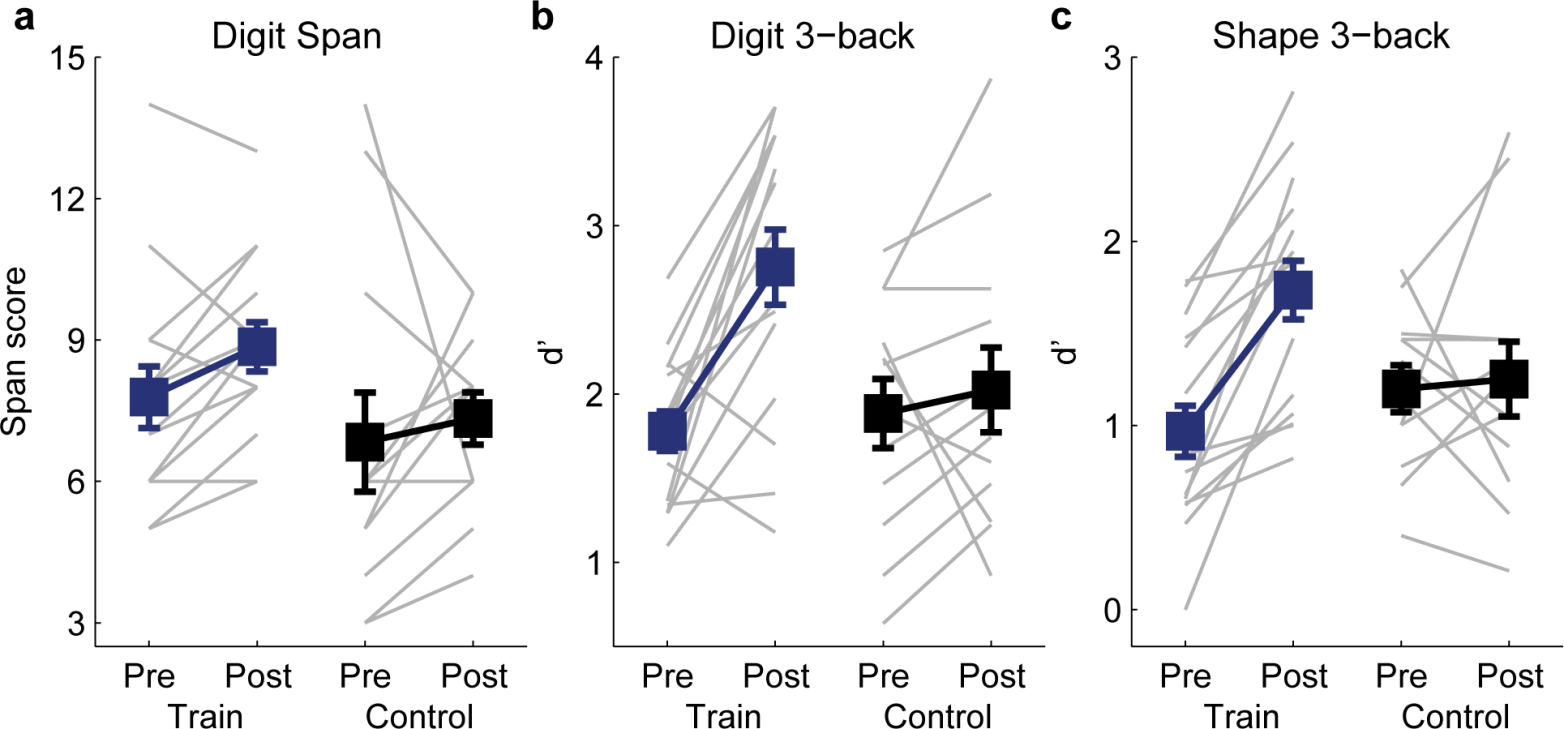
Transfer from Tone n-back to other WM tasks. Plotted are individual (grey lines) and group mean (filled squares) pre- and post-training performance of the trained participants (N = 17; blue) and controls (N = 12; black) on (a) backward Digit Span, (b) digit 3-back, and (c) shape 3-back.

To assess the specificity of WM learning to stimulus type, we included (for 15 of the WM training participants and 12 controls) in the pre- and post-training tests three other WM tasks: backward Digit Span, 3-back with spoken digits, and 3-back with random shapes [26]. There was no group difference before training on any of these tasks (see Table C in S1 File). Tone n-back training did not improve performance on backward (verbal) Digit Span (repeated-measures ANOVA, group by test interaction: F_1,24_ = 0.32, p = 0.58; Fig 5A), corroborating previous reports [26, 31] that n-back and span tasks measure different aspects of WM. However, performance on both the (verbal) digit (F_1,24_ = 7.72, p = 0.010; Fig 5B) and (visual) shape (F_1,25_ = 9.17, p = 0.006; Fig 5C) 3-back tasks improved significantly, demonstrating a transferable benefit of training across stimulus modality.

**Fig 6:**
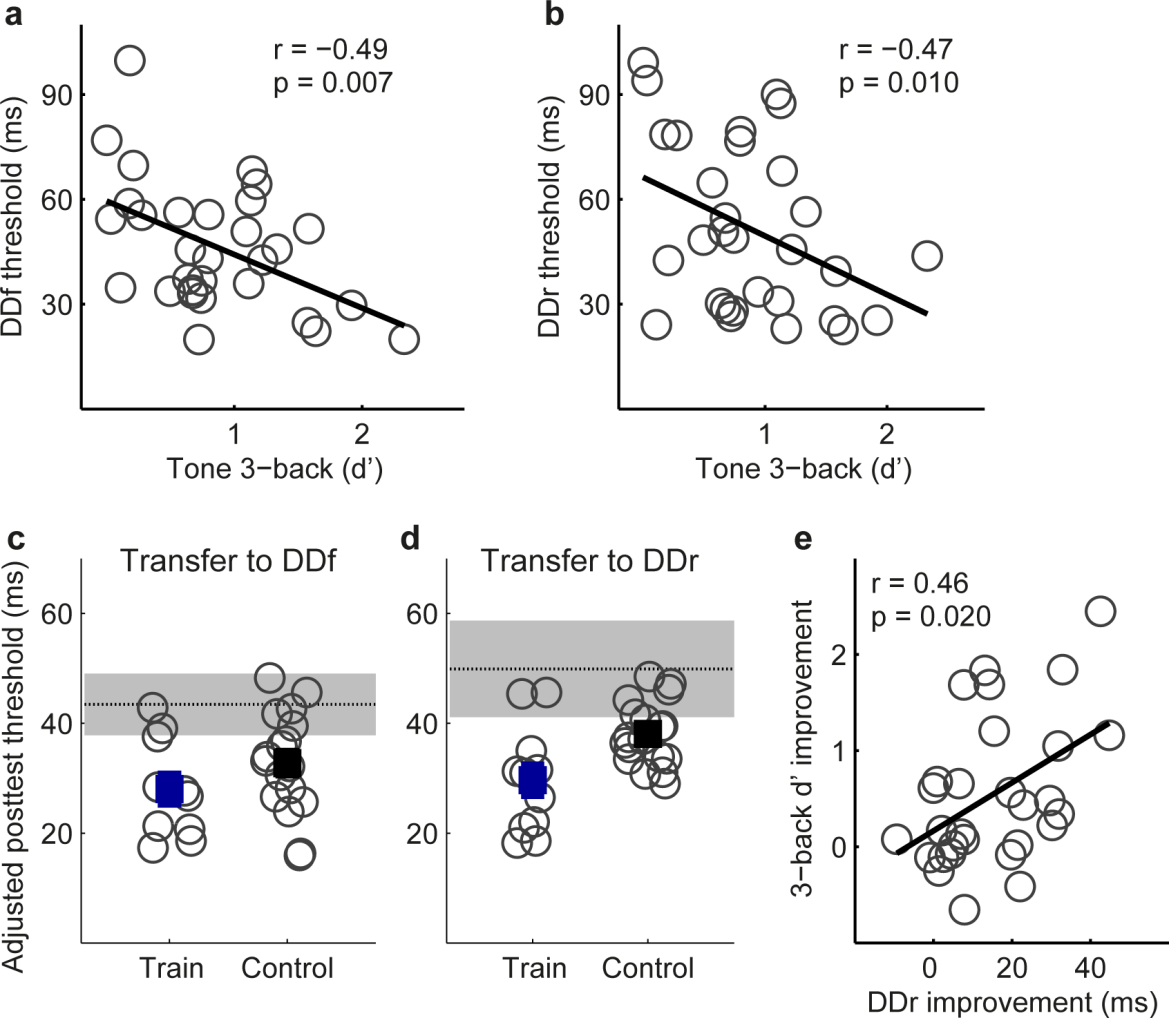
Transfer from Tone n-back to tone duration discrimination. (a, b) Correlation of DDf and DDr thresholds with Tone 3-back performance before training. (c, d) Individual (grey circles) and group mean (filled squares) posttest thresholds of the trained participants (N = 11) and controls (N = 15) on DDf and DDr, adjusted for differences in pretest thresholds. The horizontal dotted line and the grey patch indicate mean pretest threshold and 95% confidence interval. (e) Correlation of Tone 3-back and DDr improvements.

To assess the scope of perceptual performance that can benefit from WM learning, we also examined transfer of WM training (for 11 of the 17 WM training participants and 15 new controls) to a tone duration discrimination (DD) task. The DD task was similar to the FD task, but required comparison of tone duration instead of frequency. Before training, there was no group difference for DD or WM performance (see Table C in S1 File). WM performance was correlated with DD threshold both when the standard tone was fixed (DDf; Fig 6A) and when the standard tone was roved in frequency (DDr; Fig 6B). The WM training participants improved more than controls on the DDr condition(F_1,24_ = 18.03, p < 0.001; Fig 6D), but not on the DDf condition (F_1,24_ = 0.815, p = 0.38; Fig 6C). Similar to the case of WM transfer to FD (Fig 4C), this difference was related to the rapid learning achieved by controls on the DDf, but not the DDr, condition, indicating that WM training benefited auditory discrimination learning only when the standard stimulus was roved. WM improvement did not correlate with the change in the influence of stimulus variability (improvement in DDr–DDf; r = 0.04, p = 0.84), possibly because roving the standard frequency, an attribute irrelevant to the DD task, had no impact on performance (t_26_ = -1.05, p = 0.30). However, WM improvement was correlated with DDr improvement (Fig 6E). Thus, although the Tone n-back task focuses on frequency memory, the perceptual benefit of WM training was not limited to frequency comparisons.

### Training DDr did not improve WM

**Fig 7:**
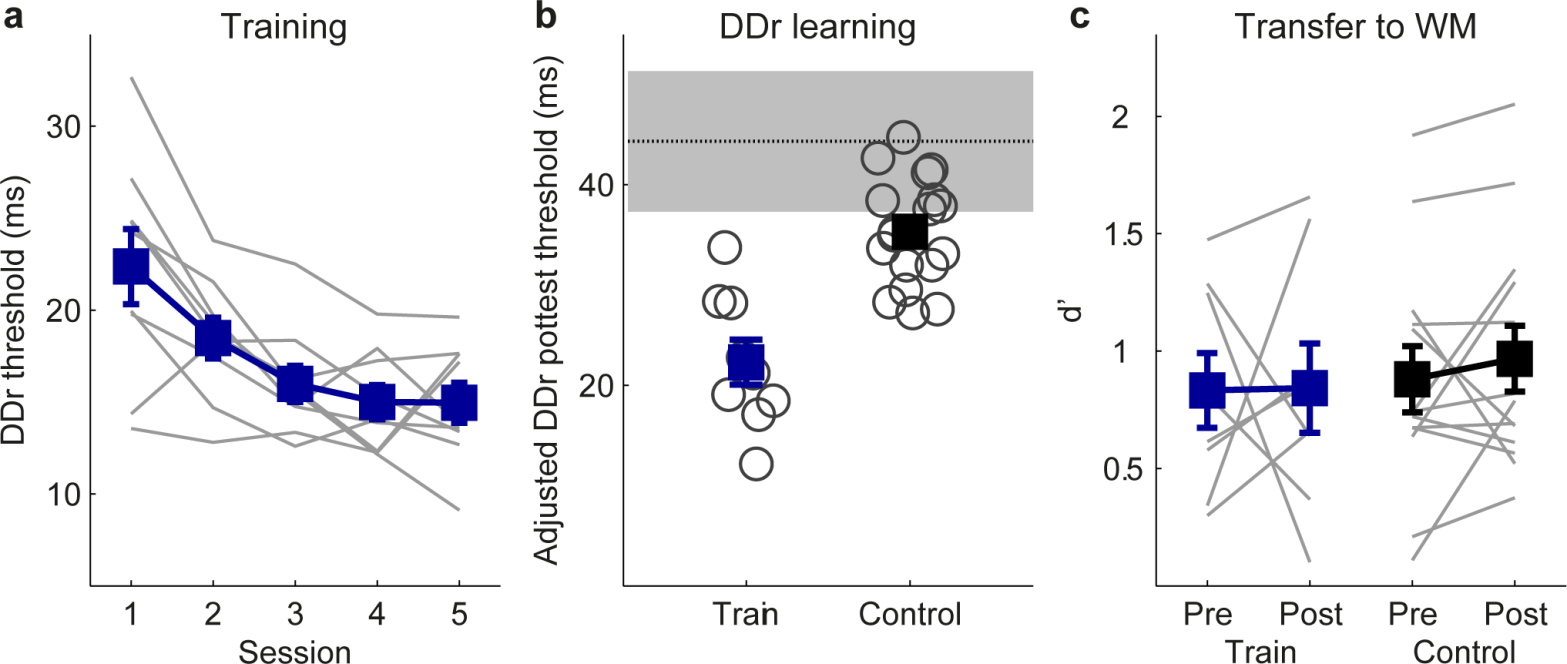
DDr training and transfer to WM. Plotted are individual (grey lines or circles) and group mean (filled squares) performance of the trained participants (N = 9) and controls (N = 15) (a) during training, (b) after training on DDr, adjusted for differences in pretest thresholds, and (c) before and after training on Tone 3-back.

Does auditory discrimination training with variable stimuli always lead to WM improvement? We proposed that FDr training improved WM because the frequency roving forced the participants to constantly update memory representation of the standard. One prediction of this proposal is that training with a variable stimulus would not improve WM if the variations were irrelevant to the task and hence not needed to be stored and updated in WM. To test this predication, we trained a new group of participants (N = 9) on DDr for five sessions of 600 trials. They were compared to the same control group (N = 15) used to test WM transfer to DDr. Before training, there was no group difference for DDf or Tone 3-back (see Table D in S1 File), but there was a trend for the training group to be better for DDr (F_1,22_ = 3.46, p = 0.076). DDr threshold decreased significantly over training (F_4,32_ = 11.10, p < 0.001; Fig 7A). To take into account possible group difference in starting DDr performance, we conducted ANOVA on posttest threshold using pretest threshold as a covariate. The trained participants performed significantly better than controls on DDr after training (F_1,21_ = 28.6, p < 0.001; Fig 7B). However, there was no group difference on Tone 3-back (F_1,22_ = 0.05, p = 0.82; Fig 7C). Thus, training DDr did not benefit WM, even though performance on the DDr task did benefit from WM training, consistent with our prediction that discrimination training with variable stimuli improves WM only if variations are relevant to the trained task and hence engage WM updating.

## General Discussion

We demonstrated that 1) auditory discrimination is related to WM updating, 2) WM training can improve auditory discrimination and, 3) auditory discrimination training can improve WM, but only with stimulus variations along the task relevant dimension. These results fit the predictions of our proposal that WM mediates across-trial stimulus interaction in auditory discrimination and contributes to discrimination learning when training with varied standard stimuli.

### Nature of the mutual transfer

We suggest that the mutual transfer between FD and Tone n-back reflects improvement by both types of training of a common WM process involved in both tasks, rather than improvement of sensory processing. Four lines of evidence converge on this conclusion. First, the tones used in the n-back task were perceptually salient, differing from each other by at least one ‘equivalent rectangular bandwidth’ (ERB) [25]. Such tones are easily discriminated by normal hearing listeners. Performance of Tone n-back tasks is thus unlikely to be influenced by further refinement of sensory acuity. Second, while discrimination performance improved following both FDf and FDr training, only FDr training improved WM performance. We cannot conceive how roving the standard would promote sensory improvements. In contrast, there are reasons to believe stimulus roving would encourage learning of cognitive processes, as outlined below. Third, the training results related WM to the ability to adapt to a varying sensory environment. It was improvement in this ability, rather than in sensory performance *per se*, that transferred to and from WM. Fourth, Tone n-back learning transferred across stimulus type (tone vs. digits) and modality (auditory vs. visual), and to a perceptual task that does not involve frequency comparison (duration discrimination), indicating that learning occurred at a supramodal level, beyond the processing of basic sound features. There is a possibility that Tone n-back learning actually consisted of two types of improvements that transferred differently. Namely, improvement due to familiarity of material transferred to perceptual tasks with tones (FDr and DDr; lack of transfer to FDf could be due to ceiling effect), while improvement in mental operations transferred to other n-back tasks. We regard this possibility as unlikely because transfer of familiarity should be mutual, but only one out of the three auditory discrimination tasks (FDr, FDf, and DDr) transferred to Tone n-back. The possibility could be directly tested in the future by examining whether n-back learning with non-tone materials (e.g., spoken digits or shapes) transferred to auditory discrimination with tones. We are aware of only one other study of transfer between perceptual and cognitive learning [35]. In that study, older adults trained on a visual discrimination task showed improved WM. However, WM was measured using perceptually challenging stimuli. Transfer was eliminated when the perceptual difficulty of the WM task was matched before and after training, indicating that the transferred skill was sensory. The visual training task was discrimination of expansion or contraction of Gabor patterns with varied colors and orientations. This task was comparable to the DDr task in the current study in that the stimulus variations were along task-irrelevant dimensions and did not require updating by WM. In this light, the lack of WM improvement following the visual-discrimination training was compatible with our proposal. The study reported here thus provides the first evidence that perceptual training can improve WM skills and that perceptual performance can benefit from WM training. We suggest that such mutual transfer is based on the involvement of WM processes in auditory discrimination.

### The role of WM in auditory discrimination

The results suggest that the extent and pattern of WM involvement in auditory discrimination depend on stimulus variability. When stimulus variability is low (e.g., with a fixed standard), WM can be used to recognize and utilize stimulus repetition to form an enhanced representation of the standard [36] that is more reliable and precise than can be attained from a single stimulus presentation (a 'perceptual anchor'`, [18]). In doing so, the system would eliminate the need to maintain individual representations of each incoming stimulus and reduce the demand on executive functions such as memory updating. Discrimination performance is limited by the precision of the perceptual anchor, and is thus unlikely to challenge WM updating during training or to benefit from WM training. When stimulus variability is high (e.g., with a roved standard), comparisons along the varied stimulus dimension have to be made “online” using the most recent standard. This requires a WM representation of the current standard to be maintained against the intrusion of old representations. The need for WM updating presents a performance-limiting source of internal noise for discrimination, evidenced by the elevation of FD threshold for a roved standard. However, comparisons along the non-varied dimension were not influenced, as indicated by the similar threshold of DDf and DDr. The differential influence of frequency roving on frequency and duration discrimination suggests that different stimulus features can be separately stored and updated in WM. This separation was confirmed by the lack of transfer from DDr learning to WM for frequency.

Baddeley’s classic model of WM [15] consists of two slave storage systems, a phonological loop for verbal information and a visuospatial sketchpad for non-verbal visual information, regulated by the same central executive system. For tonal stimuli, WM representations could be disrupted by other tones, but less so by verbal stimuli [37], leading to the suggestion that pitch memory is an additional storage system [38]. The three types of stimuli we compared in our examination of WM learning specificity (spoken digits, non-descriptive visual shapes, tones) correspond to these three storage systems. Transfer of training from Tone n-back to both the spoken digit and visual shape n-back tasks suggests that, within the scope of the model, n-back training improved the central executive. Likewise, two different auditory discrimination features benefited from WM training, consistent with performance limited by a feature-independent central executive system. As an interesting side note, WM updating captures significant individual variance in intelligence [31, 39]. Its role in auditory discrimination thus supports a recent proposal that the long observed correlation between auditory discrimination and intelligence [40, 41] is mediated by WM [42].

An alternative to the WM account is that the differential transfer from auditory discrimination to WM was a result of variation in training amount. The amount of training needed to produce perceptual learning varies considerably for different tasks [2, 43]. In the current study, discrimination training continued until learning reached asymptote, a common practice in perceptual learning studies (e.g., [44]), leading to different amounts of training across tasks (1600 trials for FDf, 3600 trials for FDr, and 3000 trials for DDr). While we demonstrated significant learning for the FDf and DDr tasks that did not transfer to Tone n-back, there remains a possibility that transfer might emerge with further training. However, the current literature on perceptual training does not support such a possibility. To date, there is no report of influence of training amount on across-task transfer, and the limited evidence on within-task transfer to untrained stimuli present a mixed picture: increasing training has been shown both to reduce [45] and to enhance [46] transfer. Note that in the latter case [46], further training only increased the magnitude of improvement on a previously transferrable condition (accompanied by increased learning), without generating transfer to novel conditions. Moreover, while the amount of perceptual training varies from minutes to weeks and even months, there is no report of trend of broader transfer for tasks requiring more extensive training (for review, see [1, 2]). Instead it has been suggested that the initial phase of learning is more transferrable while subsequent learning is more specific [3, 47]. All taken together, we consider it very unlikely that difference in training amount (by merely 600 trials or 20% between DDr and FDr) could account for the distinctive difference in transfer patterns of these tasks.

### Implications for learning, transfer and applications

This study carries some general implications for our view of learning. By demonstrating that WM learning can result from perceptual training, it extends the current view of perceptual learning as change in the encoding or decoding of sensory information and supports the idea that perceptual learning involves changes at multiple, sensory as well as cognitive processing stages [48]. This idea has received some support from neural imaging studies. For example, training on visual shape identification has been reported to result in large-scale changes throughout the visual pathway as well as in anterior and parietal cortical regions associated with attention control [49]. Under this new light, when training a given perceptual skill, what is learned (and hence how learning transfers) is not fixed, but a result of the specific training condition. The conventional repetitive training with a single standard stimulus may encourage refined neural representation of features of the trained standard or enhanced readout of that representation by the decision making unit, leading to the numerous observations of highly specific learning (for review, see [1, 2]). As repetitive exposure to a single stimulus is rarely encountered in the natural environment, the conclusions drawn from laboratory training, such as high stimulus specificity, may not apply to realistic situations. In contrast, the more complex training paradigms used in recent reports of enhanced transfer [7, 10, 11] contained variable training stimuli or tasks, which may increase cognitive engagement and encourage cognitive learning. As cognitive learning is by nature less dependent on stimulus specifics, such training would afford greater transfer.

Cognitive training has been largely directed towards improving cognitive skills that are broadly applicable [50]. However, training core cognitive skills by repetitive practice with adaptive difficulty is relatively new. Whether the training benefits transfer within the cognitive domain is still under debate [51, 52], and the possibility of transfer to perceptual performance has not previously been raised. The current results demonstrate this possibility. Those seeking to improve perceptual performance may thus consider directly training relevant cognitive skills, and those seeking to improve cognitive skills should be aware that such improvements could occur as a result of perceptual exercises. This view also supports the practice, and results, of mixed training of cognitive and perceptual tasks [53].

Finally, the utility of training is often judged by how ‘far’ the training benefits transfer. Transfer is far if the tested task is unrelated to or very different from the trained one, and near if the tested task is closely related [50]. An implicit assumption of this view is that near transfer should be a prerequisite for far transfer [52]. However, we found a closer relation of WM updating with fine auditory discrimination than with WM span, reflected both in covariance of individual differences and in transfer of learning. In this context, some observations of learning specificity may be due to the use of inappropriate transfer tasks. Supporting this idea, visual learning that did not transfer across task or stimulus configurations was shown to transfer across both dimensions provided a common spatial axis for perceptual judgments was used [54]. We therefore advocate that task relations based on behavioral (as here) or neuroimaging (e.g., [23]) evidence, rather than domain similarities, be used to evaluate transfer distance and training utility.

## Supporting Information

S1 File Pre-training performance with statistics (Table A to D) and main effects of group by test ANOVA (Table E to H) for each training and transfer experiment. (DOCX)
