# Supplementary material for: Auditory Discrimination Learning: Role of Working Memory
Source: PLoS One. 2016 Jan 22;11(1):e0147320. doi: 10.1371/journal.pone.0147320 (PMC4723131; doi:10.1371/journal.pone.0147320)
Supplement: S1 File — (DOCX) [file pone.0147320.s001.docx]

**S1 File. Pre-training performance and additional statistics.**

**Table A. Pre-training performance (mean ± SD) and statistics for the FD training experiment**

| **Condition** | **FDf (log_10_(%Hz))** | **FDr (log_10_(%Hz))** | **Tone 3-back d’** |
| --- | --- | --- | --- |
| **FDf training (N=36)** | 0.31 ± 0.85 | NA | 0.90 ± 0.58 |
| **FDr training (N=24)** | 0.32 ± 0.49 | 0.73 ± 0.45 | 0.81 ± 0.58 |
| **Control (N=36)** | 0.42 ± 0.48 | 0.72 ± 0.49 | 0.99 ± 0.63 |
| **Group effect (ANOVA)** | F_2,93_ = 0.312  p = 0.73 | F_1,58_ = 0.000  p = 0.99 | F_2,93_ = 0.620  p = 0.54 |

**Table B. Pre-training performance (mean ± SD) and statistics for the WM training experiment**

| **Condition** | **FDf (log_10_(%Hz))** | **FDr (log_10_(%Hz))** | **Tone 3-back d’** | **Tone 2-back d’** |
| --- | --- | --- | --- | --- |
| **WM training (N=17)** | 0.34 ± 0.43 | 0.77± 0.37 | 0.96 ± 0.61 | 1.39 ± 0.91 |
| **Control (N=36)** | 0.42 ± 0.48 | 0.72 ± 0.49 | 0.99 ± 0.63 | 1.68 ± 0.53 |
| **Group effect (ANOVA)** | F_1,51_ = 0.380  p = 0.54 | F_1,51_ = 0.134  p = 0.72 | F_1,51_ = 0.844  p = 0.36 | F_1,27_ = 0.926  p = 0.34 |

**Table C. Pre-training performance (mean ± SD) and statistics for WM transfer to other tasks**

| **Condition** | **Digit Span** | **Digit 3-back d’** | **Shape 3-back d’** | **DDr (ms)** | **DDf (ms)** |
| --- | --- | --- | --- | --- | --- |
| **WM training (N=15^a^)** | 7.78 ± 2.46 | 1.78 ± 0.45 | 0.97± 0.54 | 61.9 ± 18.1 | 46.9 ± 12.7 |
| **WM control (N=12)** | 6.83 ± 3.63 | 1.88 ± 0.71 | 1.20 ± 0.44 |  |  |
| **DD control (N=15)** |  |  |  | 48.6 ± 24.0 | 44.7± 21.3 |
| **Group effect (ANOVA)** | F_1,24_ = 0.628  p = 0.44 | F_1,25_ = 0.220  p = 0.64 | F_1,25_ = 1.42  p = 0.24 | F_1,24_ = 2.38  p = 0.14 | F_1,24_ = 0.095  p = 0.76 |

**^a^11 tested on DD conditions**

**Table D. Pre-training performance (mean ± SD) and statistics for the DDr training experiment**

| **Condition** | **Tone 3-back d’** | **DDf (ms)** | **DDr (ms)** |
| --- | --- | --- | --- |
| **DDr training (N=9)** | 0.72 ± 0.52 | 38.1 ± 16.3 | 31.9 ± 12.3 |
| **Control (N=15)** | 0.75 ± 0.48 | 44.7 ± 21.3 | 48.2 ± 24.2 |
| **Group effect (ANOVA)** | F_1,22_ = 0.018  p = 0.89 | F_1,22_ = 0.641  p = 0.43 | F_1,22_ = 3.46  p = 0.076 |

**Table E. Main effects of group by test ANOVA for the FD training experiment**

| **Condition** | **FDf** | **FDr** | **Tone 3-back** |
| --- | --- | --- | --- |
| **Effect of group** | F_1,70_ = 9.43  p = 0.003 | F_1,70_ = 2.15  p = 0.15 | F_2,90_ = 0.195  p = 0.82 |
| **Effect of test** | F_1,70_ = 148.3  p < 0.001 | F_1,70_ = 141.8  p < 0.001 | F_1,90_ = 1.05  p = 0.31 |

**Table F. Main effects of group by test ANOVA for the WM training experiment**

| **Condition** | **FDf** | **FDr** | **Tone 3-back** | **Tone 2-back** |
| --- | --- | --- | --- | --- |
| **Effect of group** | F_1,49_ = 1.07  p = 0.31 | F_1,50_ = 0.502  p = 0.48 | F_1,51_ = 8.18  p = 0.006 | F_1,27_ = 0.201  p = 0.66 |
| **Effect of test** | F_1,49_ = 66.0  p < 0.001 | F_1,50_ = 118.7  p < 0.001 | F_1,51_= 27.5  p < 0.001 | F_1,27_ = 18.2  p < 0.001 |

**Table G. Main effects of group by test ANOVA for WM transfer to other tasks**

| **Condition** | **Digit Span** | **Digit 3-back** | **Shape 3-back** | **DDr** | **DDf** |
| --- | --- | --- | --- | --- | --- |
| **Effect of group** | F_1,24_ = 2.04  p = 0.17 | F_1,25_ = 1.65  p = 0.21 | F_1,25_ = 0.433  p = 0.52 | F_1,24_ = 0.523  p = 0.48 | F_1,24_ = 0.042  p = 0.84 |
| **Effect of test** | F_1,24_ = 2.37  p = 0.14 | F_1,25_ = 13.8  p = 0.001 | F_1,25_ = 12.1  p = 0.002 | F_1,24_ = 61.1  p < 0.001 | F_1,24_ = 18.1  p < 0.001 |

**Table H. Main effects of group by test ANOVA for DDr training experiment**

| **Condition** | **Tone 3-back** | **DDf** | **DDr^a^** |
| --- | --- | --- | --- |
| **Effect of group** | F_1,22_ = 0.083  p = 0.78 | F_1,22_ = 2.10  p = 0.16 | NA |
| **Effect of test** | F_1,22_ = 0.84  p = 0.37 | F_1,22_ = 23.0  p < 0.001 | NA |

^a^group by test ANOVA was not conducted due to group difference in pretest threshold
